# Elucidation of glutamine lipid biosynthesis in marine bacteria reveals its importance under phosphorus deplete growth in *Rhodobacteraceae*

**DOI:** 10.1038/s41396-018-0249-z

**Published:** 2018-08-14

**Authors:** Alastair F. Smith, Branko Rihtman, Rachel Stirrup, Eleonora Silvano, Michaela A. Mausz, David J. Scanlan, Yin Chen

**Affiliations:** School of Life Sciences, University of Warwick, Coventry, CV4 7AL UK

**Keywords:** Environmental microbiology, Microbial ecology

## Abstract

Marine microorganisms employ multiple strategies to cope with transient and persistent nutrient limitation, one of which, for alleviating phosphorus (P) stress, is to substitute membrane glycerophospholipids with non-P containing surrogate lipids. Such a membrane lipid remodelling strategy enables the most abundant marine phytoplankton and heterotrophic bacteria to adapt successfully to nutrient scarcity in marine surface waters. An important group of non-P lipids, the aminolipids which lack a diacylglycerol backbone, are poorly studied in marine microbes. Here, using a combination of genetic, lipidomics and metagenomics approaches, we reveal for the first time the genes (*glsB*, *olsA*) required for the formation of the glutamine-containing aminolipid. Construction of a knockout mutant in either *glsB* or *olsA* in the model marine bacterium *Ruegeria pomeroyi* DSS-3 completely abolished glutamine lipid production. Moreover, both mutants showed a considerable growth cost under P-deplete conditions and the *olsA* mutant, that is unable to produce the glutamine and ornithine aminolipids, ceased to grow under P-deplete conditions. Analysis of sequenced microbial genomes show that *glsB* is primarily confined to the *Rhodobacteraceae* family, which includes the ecologically important marine *Roseobacter* clade that are key players in the marine sulphur and nitrogen cycles. Analysis of the genes involved in glutamine lipid biosynthesis in the *Tara* ocean metagenome dataset revealed the global occurrence of *glsB* in marine surface waters and a positive correlation between *glsB* abundance and N* (a measure of the deviation from the canonical Redfield ratio), suggesting glutamine lipid plays an important role in the adaptation of marine *Rhodobacteraceae* to P limitation.

## Introduction

Bacterial membranes form the barrier separating bacteria from their surrounding environment, with membrane lipids being an essential component of this structure. Our knowledge of bacterial lipids is predominantly derived from studies of model organisms, e.g., *Escherichia coli*, which is primarily composed of several glycerophospholipids, phosphatidylethanolamine, phosphatidylglycerol and a small amount of cardiolipin [1]. However, beyond *Escherichia coli*, we now know that a range of lipids are found in bacterial membranes, including phosphorus (P)-containing glycerophospholipids but also P-free lipids that are composed of a diacylglycerol backbone. The latter include betaine lipids, e.g., diacylglycerol-*N,N,N*-trimethylhomoserine (DGTS), sulfolipids, e.g., sulfoquinovosyl-diacylglycerol and glycolipids, e.g., monoglycosyl-diacylglycerol (MGDG) and glucuronic acid diacylglycerol (GADG) [2]. In the marine environment, it is well established that P availability significantly affects lipid composition in marine phytoplankton as well as cosmopolitan marine heterotrophic bacteria [3–5]. In fact, several lipid surveys (environmental lipidomics) have been carried out in marine waters and the ratio of non-P lipids to phospholipids is a useful marker for detecting P-stress in natural microbial communities (e.g., [3, 4, 6–8]).

An important, yet poorly studied group of P-free lipids are the amino-acid containing lipids [9]. Unlike the aforementioned lipids, these aminolipids do not contain a diacylglycerol backbone. Instead, these aminolipids contain an amino acid head group linked to a fatty acid (usually a β-hydroxy fatty acid) through an amide bond. Arguably, the best studied aminolipid is the ornithine lipid which contains the non-proteinogenic amino acid ornithine as the head group. Ornithine lipids have been widely reported in bacteria [10], being found in marine surface water lipidomic surveys [6] and the abundant marine heterotroph SAR11 [5]. Biosynthesis of ornithine lipids is carried out either by a two-step process using two acyltransferases encoded by the *olsB* and *olsA* genes or by the bifunctional fusion protein OlsF [11]. Other amino acid head groups found in bacterial aminolipids include glutamine, lysine and serine [9, 12]. However, these aminolipids have not been reported in environmental lipidomics surveys of marine waters and the metabolic pathways underpinning the biosynthesis of non-ornithine aminolipids are unknown.

Here, we report the identification and characterisation of glutamine lipid in members of the cosmopolitan marine *Roseobacter* clade, a group of *Alphaproteobacteria* that are abundant in coastal marine waters and play important roles in the biogeochemical cycling of S and N (see reviews by [13, 14] and references therein). We reveal the genes required for glutamine lipid biosynthesis and demonstrate that this glutamine lipid is predominantly found in marine *Roseobacter* and closely related members of the *Rhodobacteraceae* family. Moreover, this lipid appears to be important for maintaining normal cellular function during P deplete conditions.

## Materials and methods

### Bacterial strains, media and cultivation conditions

Bacterial strains, plasmids and PCR primers used in this study are listed in Suppl. Table S2. Marine bacteria used in this study were cultivated using either marine broth (Difco Marine Broth 2216 (Becton, Dickinson and Company, Sparks, MD, USA), ½ YTSS (2 g/L yeast extract, 1.25 g/L peptone, 20 g/L sea salts, Sigma-Aldrich), or a defined MAMS medium [15]. *E. coli* strains were routinely cultivated in lysogeny broth with appropriate antibiotics.

### Construction of mutants of *Ruegeria pomeroyi*

Marker-exchange mutagenesis was used to construct the Δ*glsB* (SPO2489) and Δ*olsA* (SPO1980) mutants of *R. pomeroyi* DSS-3 [16]. Briefly, primers were designed to PCR amplify 500–700 base pair regions either side of the target gene. These two fragments, together with a gentamicin-resistant cassette, were cloned into vector pK18*mobsacB* in *E. coli* S17-1λ*pir*. The construct was then conjugated to *R. pomeroyi* DSS-3. Transformants were selected on marine sea salt agar plates supplemented with 10 mM glucose, 2 mM glycine betaine and 10 μg/mL gentamicin. Double-crossover deletion mutants were selected for their sensitivity to kanamycin (50 μg/mL). The mutants were confirmed by PCR and subsequent sequencing.

To compare the growth of the Δ*glsB* and Δ*olsA* mutants with wild-type *R. pomeroyi* DSS-3, cells were grown in defined MAMS medium with either 0.5 mM or 5 mM phosphate in three biological replicates. Bacterial growth was quantified by measuring optical density (OD) at 540 nM at regular intervals. Alkaline phosphatase activity was measured prior to the collection of samples for lipid analysis. Pairwise comparisons of the growth rates of each strain grown at high and low phosphate concentrations, as well as comparisons of the growth rates between strains grown at the same phosphate concentration, were made using a Student’s *t*-test.

### Intact polar lipid extraction and analysis

Lipids from bacterial cultures were extracted using a modified Folch extraction method [17]. Briefly, 1 mL of culture with an OD_540_~0.5 was collected. The cells were pelleted by centrifugation and re-suspended in 0.5 mL of LC–MS grade methanol (Sigma-Aldrich) in a 2 mL glass Chromacol vial (Thermo Scientific). Lipid extraction was carried out using chloroform-methanol. Solvent-extracted lipids were dried under nitrogen gas using a Techne sample concentrator (Staffordshire, UK) and lipid pellets were re-suspended in 100 µL 1:1 (v/v) chloroform: methanol and 900 µL acetonitrile. These samples were then analysed by LC-MS employing a Dionex 3400RS HPLC, coupled to an AmazolSL quadrupole ion trap MS (Bruker Scientific) via an electrospray ionisation interface. Separation of lipids in HPLC was carried out using a BEH amide XP column (Waters). The column was maintained at 30 °C, with a flow rate of 150 μL min^−1^. Samples were run on a 15-min gradient from 95% (v/v) acetonitrile to 28% (w/v) 10 mM ammonium acetate pH 9.2, with 10 minutes equilibration between samples. Each sample was analysed in both positive and negative ionisation modes. Data analyses were carried out using the Bruker Compass software package, using DataAnalysis for peak identification and characterization of lipid class, and QuantAnalysis for quantification of the relative abundance of aminolipids to phosphatidylethanolamine.

### Alkaline phosphatase activity assay

Alkaline phosphatase activity was used to assess whether cultures were stressed for P availability using para-nitrophenol phosphate (pNPP) as the substrate. A stock solution of 10 mM pNPP (Sigma-Aldrich) was prepared in 10 mM Tris-HCl pH 7.0. 900 µL aliquots of cell culture were added to 100 µL pNPP stock solution to obtain a final pNPP concentration of 1 mM. Control incubations were set up without the cultures in parallel. Formation of the yellow-coloured para-nitrophenol (pNP) was recorded by monitoring absorbance at 405 nM using a BioRad imark microplate reader. A calibration curve was made using pNP standards (Sigma-Aldrich) in the range between 10 µM–2 mM.

### Phylogenetic and metagenome/metatranscriptome analyses

Phylogenetic analysis of 16S rRNA genes from *Rhodobacteraceae* was carried out using the full length 16S rRNA gene retrieved from the IMG database (https://img.jgi.doe.gov/). GlsB and OlsB sequences were retrieved from the IMG database using BLASTP searches using SPO2489 and SPO1980 as the query sequence, respectively, with an *e*-value cut-off of 10^−5^. The retrieved homologues were then manually inspected using the neighbourhood view in IMG for the presence of *bamE* and *olsA*, in the neighbourhood of *glsB* and *olsB*, respectively. Sequence alignment was performed using Muscle and phylogenetic analyses were performed with RaxML with 100 bootstrap replicates [18].

To search for GlsB, OlsB and OlsF homologues in the *Tara* metagenome datasets, a single Hidden Markov model profile was constructed using Hmmer3 with an *e*-value cut-off of 10^−5^. The reference sequence was chosen to represent OlsB, GlsB and OlsF whose functions had been validated experimentally. This reference alignment was used to construct a maximum likelihood phylogenetic tree using RaxML with 100 bootstrap replicates. In order to classify the sequences retrieved from the *Tara* metagenomes by Hmm search, their maximum likelihood placement onto this reference phylogeny was determined using pplacer [19]. The abundance of each gene in the *Tara* metagenomes was standardised to RecA abundance retrieved using an Hmm search using the same *e*-value cut-off.

**Fig. 1:**
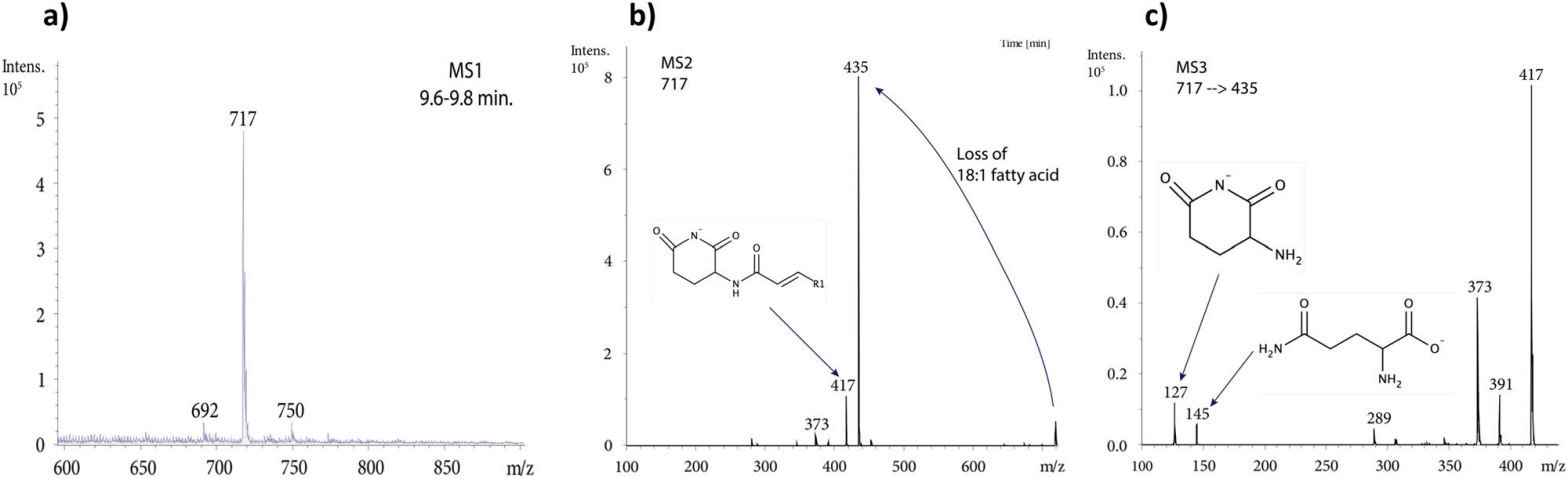
Characterisation of glutamine lipid from *Ruegeria pomeroyi* DSS-3 cultures by mass spectrometry in negative ion mode. **a** Mass spectrum showing molecular ions detected in the peak eluting between 9.6 and 9.8 minutes. The most abundant ion, with *m/z* 717, was selected for further fragmentation (**b, c**). After a first round (MS^2^) of fragmentation (**b)**, the major ion with *m/z* 435 was consistent with a loss of an 18:1 fatty acid. This ion was selected for MS^3^ fragmentation (**c**), which yielded diagnostic ions with *m/z* 145 and 127, corresponding to glutamate and to an ion resulting from the cyclisation of glutamate following loss of water, respectively

To search for *glsB* in metatranscriptome datasets, we used the JGI IMG metatranscriptome database, which contained 428 datasets from marine ecosystems (as of 12 June 2018). A BlastP search was carried out using GlsB from *Ruegeria pomeroyi* DSS-3 as the query sequence (SPO2489) with a stringent *e*-value cut-off of 10^−20^, which yielded 131 sequences at varying lengths (Table S1). The taxonomy of the retrieved GlsB sequences was assigned by a BlastP search against the NCBI non-redundant protein sequences and the top BlastP hit, together with sequence similarity value, *e*-value and accession number, is presented in Table S1. GlsB sequences retrieved from the metatranscriptome datasets (>140 amino acids in length) were then aligned and mapped to the GlsB/OlsB/OlsF reference tree to confirm their phylogenetic position (Figure S3).

### Statistical analysis

Linear regression was used to investigate relationships between the abundance of *olsB*, *glsB* and *olsF* genes and a measure of relative phosphate availability in the *Tara* metagenome dataset. Since many samples in the *Tara* metagenome dataset have a very low phosphate concentration, the ratio of nitrogen-to-phosphorus traditionally used in microbial ecology is problematic as the denominator is close to zero. Therefore, we chose to use the measure of the relative abundance of nitrogen-to-phosphorus introduced by Weber and Deutsch [20] where $N^ \ast = \left[ {NO_3^ - } \right] - 16\left[ {PO_4^{3 - }} \right]$. The abundance of *olsB*, *glsB* and *olsF* takes the form of the aforementioned count data retrieved from each *Tara* metagenome. In order to assess whether N* was a significant predictor of the abundance of each aminolipid synthesis gene (i.e., *olsB*, *glsB* and *olsF*), two models were compared for each gene. A base model was constructed in which the abundances of each of the microbial groups to which sequences for that gene were assigned were included as covariates (Suppl. Table S3). This was compared to the second model that was identical to the base model but with the addition of a term for N* using likelihood ratio tests. Scatterplots and generalised linear model fits showing the relationship between aminolipid synthesis gene counts and N* in the *Tara* metagenomes are shown in Suppl. Figure S4. The abundance values for each microbial group were calculated from metagenomic 16S Illumina tag data, which is available from http://ocean-microbiome.embl.de/companion.html [21, 22].

## Results

### Identification of glutamine lipids in *Ruegeria pomeroyi* DSS-3 by mass spectrometry

We have previously grown several marine *Roseobacter* strains in the laboratory in order to investigate the link between nutrient availability and lipid remodelling in these ecologically important marine bacteria [3]. When analysed by high-performance liquid chromatography (HPLC)-mass spectrometry (MS) in negative ionisation mode, these strains revealed the presence of a new lipid that consistently eluted at ~9.6 min in several *Roseobacter* strains tested, including *Ruegeria pomeroyi* DSS-3 (Fig. 1). The most abundant lipid species at ~9.6 min has a mass to charge ratio (*m/z*) of 717, corresponding to one of the most abundant lipid species previously observed in *Rhodobacter sphaeroides* [23]. In order to investigate the structure of this *m/z* = 717 ion, multiple rounds of fragmentation (MS^n^) were performed using a quadrupole ion trap MS and the sequential fragmentation patterns obtained (Fig. 1**)**. These patterns are consistent with the presence of a glutamine head group [23], indicated by two characteristic ions with *m*/*z* 145 and 127, respectively, which corresponds to glutamine after the loss of a proton and cyclisation of glutamate following loss of a water molecule (Fig. 1).

**Fig. 2:**
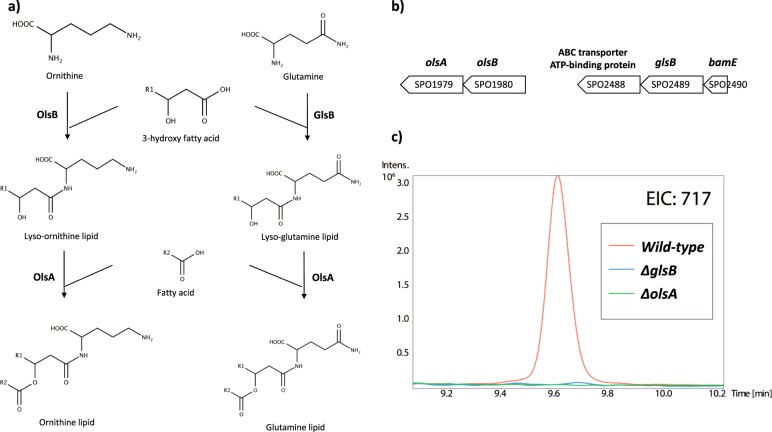
**a** Proposed pathway for the biosynthesis of glutamine lipid, in comparison to ornithine lipid biosynthesis, in *R. pomeroyi* DSS-3. The first step is carried out by an *N*-acetyltransferase encoded by *glsB* and *olsB*, respectively and lysolipid intermediates are formed. The second step is mediated by an *O-*acetyltransferase encoded by *olsA* by esterification of a second fatty acid to the hydroxyl group of the lysolipid intermediates. **b** The gene neighbourhood of *olsB* (SPO1980) and *glsB* (SPO2489) in the genome of *R. pomeroyi* DSS-3. **c** Extracted ion chromatograms (EIC) obtained after analysing lipid extract from the wild-type *R*. *pomeroyi* DSS-3 and the *olsA* and *glsB* mutants by mass spectrometry in negative ion mode. Ions with mass-to-charge (*m/z*) 717 correspond to the intact mass of the glutamine lipid (Fig. 1)

### The *SPO2489* gene is required for glutamine lipid biosynthesis in *Ruegeria pomeroyi* DSS-3

Having confirmed the presence of glutamine lipid in *R. pomeroyi* DSS-3, we set out to identify the genes involved in its biosynthesis. To the best of our knowledge the presence of this aminolipid has only been previously reported in *Rhodobacter sphaeroides* [12, 23]. However, the genes underpinning glutamine lipid biosynthesis are unknown. Due to its structural similarity to ornithine lipid, which is probably the best studied bacterial aminolipid, it has been previously hypothesised that an *N*-acetyltransferase is required for the initial condensation of glutamine to a 3-hydroxy fatty acid, followed by an *O*-acetyltransferase for adding a second fatty acid [9]. In *Rhodobacter sphaeroides* and *Ensifer meliloti*, the two-step ornithine lipid biosynthesis pathway is carried out by *olsB* and *olsA*, encoding an *N*-acetyltransferase and an *O*-acetyltransferase, respectively (Fig. 2a). Close investigation of the *R. pomeroyi* DSS-3 genome allowed the identification of OlsB and OlsA, encoded by SPO1980 and SPO1979, respectively (Fig. 2b). Interestingly a second *olsB*-like gene (SPO2489) was also found in the *R. pomeroyi* DSS-3 genome, showing 29% sequence identity to OlsB. We therefore speculated that SPO2489 (hereafter designated as *glsB* for glutamine lipid synthesis) is involved in glutamine lipid biosynthesis. Because no other OlsA-like *O*-acetyltransferase was found in the *R. pomeroyi* DSS-3 genome, we suspected *olsA* was also responsible for glutamine lipid synthesis (Fig. 2a).

**Fig. 3:**
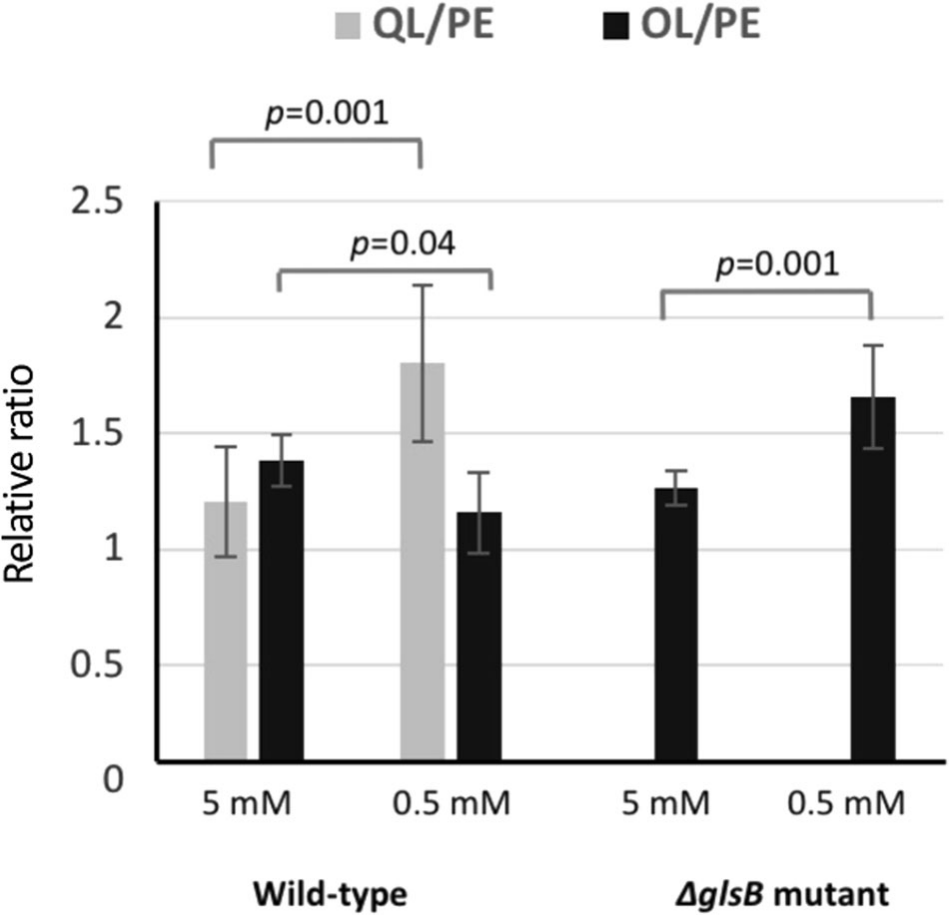
The relative abundance of glutamine lipid (QL) and ornithine lipid (OL), normalised against phosphatidylethanolamine (PE) in the wild type and the Δ*glsB* mutant under high (5 mM) and low (0.5 mM) phosphate conditions. Measurements were carried out in three biological replicates each with three technical replicates and the error bars represent standard deviation

To test the hypothesis that *glsB* and *olsA* are involved in glutamine lipid biosynthesis, we constructed marker exchange mutants using a gentamicin resistance cassette in *R. pomeroyi* DSS-3 [3, 16]. As predicted, a deletion mutant in either *glsB* or *olsA* completely abolished the formation of the glutamine lipid, as assessed by MS of membrane lipid extracts (Fig. 2c). However, whilst deletion of *glsB* did not affect the formation of ornithine lipid (data not shown), the *olsA* mutant was also unable to synthesise ornithine lipid, agreeing with our proposed biosynthetic pathway model (Fig. 2a) that *olsA* is responsible for the last step of both ornithine and glutamine lipid biosynthesis in this bacterium.

### Characterization of glutamine lipid mutants under phosphorus stress

In order to investigate the role of the glutamine lipid in *R. pomeroyi* DSS-3 in response to P availability, we used a defined marine ammonium mineral salts (MAMS) medium and compared the growth of the wild-type, *ΔolsA* and *ΔglsB* mutants, the latter two strains being unable to synthesize this glutamine lipid. A concentration of 0.5 mM phosphate was sufficient to induce P stress in this bacterium, with alkaline phosphatase activity in the wild-type in these low P grown cultures (6.25 ± 0.97 μM pNP h^−1^ OD_540_^−1^) significantly higher (*t*-test, *p* < 0.001) than wild-type cells grown in high P medium (5 mM) (0.86 ± 0.09 μM pNP h^−1^ OD_540_^−1^). When the *ΔolsA* and *ΔglsB* mutants were cultivated in high P medium, no significant difference in growth rate was observed (Table 1). However, when the mutants were cultivated in low P medium, the *ΔolsA* mutant failed to grow (Suppl. Figure S1a) and the *ΔglsB* mutant had a significantly reduced growth rate (0.077 ± 0.012 h^−1^) compared to that of the wild-type (0.096 ± 0.008 h^−1^).

**Table 1:** Growth rates of the glutamine lipid mutants compared with the wild-type *Ruegeria pomeroyi* DSS-3 at different P concentrations in a defined minimal medium

	Growth rate (h^−1^)	
	5 mM phosphate	0.5 mM phosphate
Wild-type	0.110 ± 0.008	0.096 ± 0.008
*ΔglsB* mutant	0.108 ± 0.005	0.077 ± 0.012
*ΔolsA* mutant	0.096 ± 0.012	No growth

We further analysed the lipidome of wild-type *Ruegeria pomeroyi* DSS-3 and the *ΔglsB* mutant under high (5 mM) and low phosphate (0.5 mM) conditions. Because the *ΔolsA* mutant produced neither the glutamine nor ornithine lipid and failed to grow in low phosphate medium, its lipidome was not analysed further. *Ruegeria pomeroyi* DSS-3 does not have the PlcP-mediated lipid remodelling pathway [3]. The only lipids that are made comprising glycerol-based backbones are glycerophospholipids (phosphatidylethanolamine, PE and phosphatidylglycerol, PG); DGTS, MGDG and GADG were not found. Due to the lack of available standards for aminolipids, we compared relative abundance using the ratio of glutamine lipid (QL) to PE and ornithine lipid (OL) to PE under high and low phosphate conditions (Fig. 3). This analysis showed that wild-type *Ruegeria pomeroyi* DSS-3 had a significantly elevated ratio of QL:PE under P stress conditions (*t*-test, *p* = 0.001) suggesting a substitution of PE for the glutamine lipid under these conditions. On the other hand, the OL:PE ratio did not change under P stress conditions in wild type *Ruegeria pomeroyi* DSS-3. However, in the *ΔglsB* mutant, which does not produce glutamine lipid, the OL:PE ratio significantly increased under P stress. Quantification of the change in PE in wild-type *Ruegeria pomeroyi* DSS-3 and the *ΔglsB* mutant under high and low P conditions (Suppl. Figure S1b) showed PE levels were significantly reduced under low P conditions. Taken together, our data suggests that the glutamine lipid is important for *R*. *pomeroyi* DSS-3 to maintain maximal cell growth particularly during P stress conditions and that the glutamine and ornithine lipids may be functionally interchangeable in this bacterium.

**Fig. 4:**
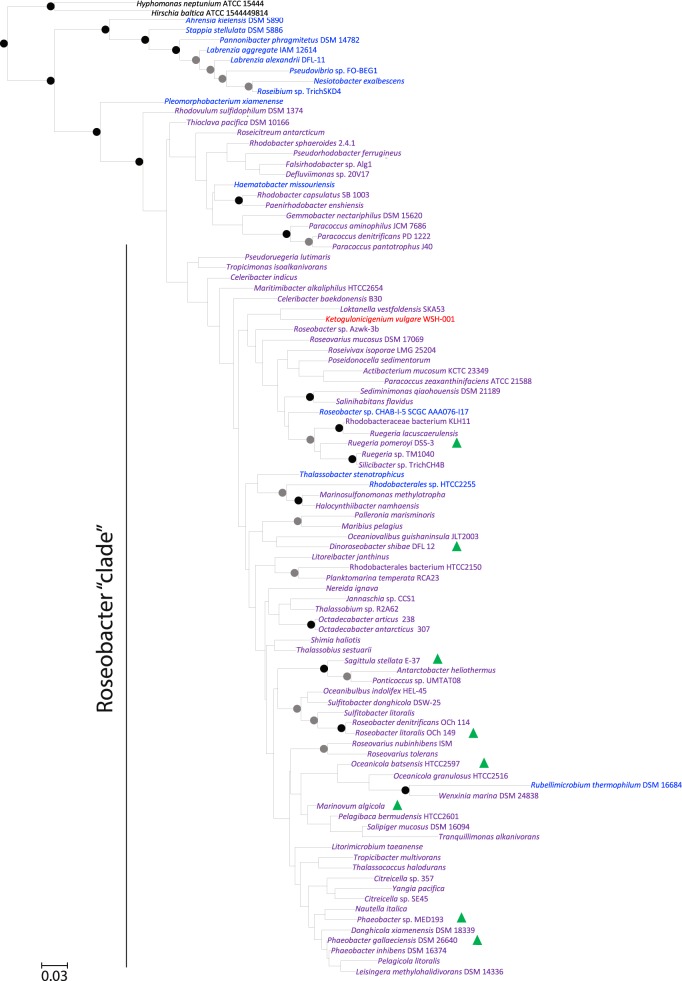
Maximum likelihood 16S rRNA gene phylogeny of *Rhodobacteraceae* with fully or partially sequenced genomes. Bootstrap support for nodes is indicated by filled circles with a black circle indicating support > 70%, a grey circle indicating 50–70% and the absence of circles indicating <50% bootstrap support. The colours indicate the presence of *olsB* alone (blue), *glsB* alone (red) or both homologues (purple) in the genome as detected by BLASTP searches using an *e*-value cut-off of 10^−5^. Strains that are verified for the production of glutamine lipid in this study by liquid chromatography-mass spectrometry are indicated by a green triangle

### *glsB* gene presence appears to be restricted to the *Rhodobacteraceae* family

Since the presence of the glutamine lipid has only previously been reported in *Rhodobacter sphaeroides* [12, 23], we set out to investigate the distribution of glutamine lipid biosynthesis potential in genome-sequenced bacteria in the integrated microbial genomes (IMG) database using *glsB* as the functional gene marker. Interestingly, this analysis indicated that *glsB* is only found in bacteria of the *Rhodobacteraceae* family. In contrast, the *olsB/olsF* genes are more widespread across bacterial phyla, including *Proteobacteria* and *Bacteroidetes*, agreeing with a previous study showing that around half of the genome-sequenced bacteria are capable of producing ornithine lipids [9]. Notably, *glsB* occurs widely in the *Rhodobacter*—*Paracoccus* group as well as the marine *Roseobacter* clade (Fig. 4), including true pelagic *Roseobacter* strains such as *Planktomarina temperata* RCA23 [24] and Rhodobacterales sp. HTCC2150 [25]. The *Rhodobacter*—*Paracoccus* group and the marine *Roseobacter* group are evolutionally related and may come from a common ancestor according to a recent phylogenomics analysis [26]. To confirm the occurrence of glutamine lipids in the *Rhodobacteraceae* we extracted membrane lipids from selected strains of the *Roseobacter* group and analysed the presence of the *m*/*z* 717 ion by mass spectrometry. We indeed found glutamine lipids present in all the cultures analysed (Fig. 4).

**Fig. 5:**
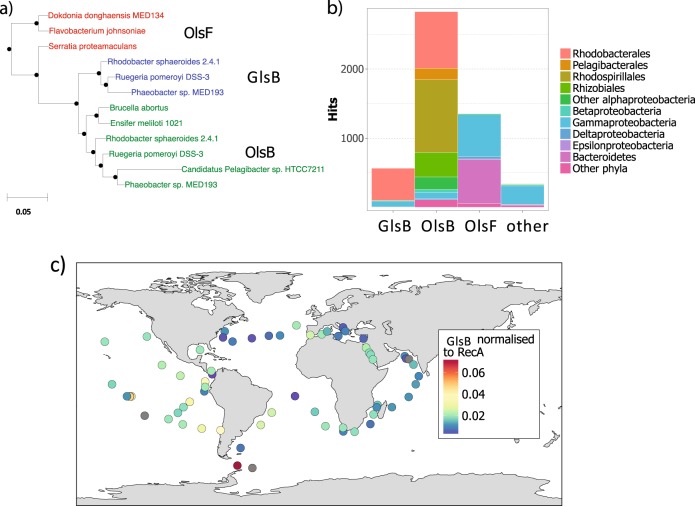
**a** Maximum likelihood phylogeny showing the evolutionary relationship between *N*-acyltransferases involved in glutamine lipid (GlsB) and ornithine lipid biosynthesis (OlsB and OlsF). **b** The number of sequences retrieved from the *Tara* metagenome data set that are assigned to each gene. Other represents environmental sequences which could not be unambiguously classified using our phylogenetic approach. **c** Global maps of the abundance of GlsB, normalised to the abundance of RecA in the *Tara* metagenome dataset. Only surface water samples (collected at 5 m depth) are shown. Grey circles indicate no sequences corresponding to that gene were detected in the sample

### Glutamine lipid biosynthesis in marine metagenomes and metatranscriptomes

In order to better understand the role of glutamine lipids in marine ecosystems, we next investigated the distribution of *glsB* (for glutamine lipid synthesis) and *olsB/olsF* (for ornithine lipid synthesis) in the *Tara* ocean metagenome data set [27]. Because GlsB and OlsB/OlsF shows significant amino acid sequence similarity, we first verified whether genes responsible for ornithine and glutamine lipid biosynthesis can be reliably separated phylogenetically using sequences from the *Rhodobacteraceae* genomes. This analysis showed that GlsB/OlsB sequences were consistently separated into two major clades (100% bootstrap support) and that these two clades were congruent with a classification based on synteny (Suppl. Figure S2). The *olsB* gene is found in the neighbourhood of *olsA* whereas *glsB* is located next to the *bamE* gene which encodes a membrane lipoprotein involved in outer membrane protein assembly [28].

To classify environmental sequences retrieved from the *Tara* metagenomes using this phylogenetic approach, an alignment of reference sequences of OlsB, GlsB and the *N*-acyltransferase domain of OlsF was created (Fig. 5a**)**. The functions of these genes have either been verified experimentally (see above, [5, 11]) or, in the case of marine strains, the strain has been shown to produce ornithine lipid or glutamine lipid. An HMMER profile was built and used as a query to search the *Tara* metagenomes (*e*-value cut-off 10^−5^). This analysis retrieved 5,097 sequences, 567 of which are classified as *glsB*. Genes encoding ornithine lipid biosynthesis are more abundant than *glsB* in these metagenomes (Fig. 5b). Phylogenetically, the majority (85%) of *glsB* genes are classified within the *Rhodobacteraceae* family in agreement with the predominant occurrence of *glsB* in genome-sequenced isolates of the *Rhodobacteraceae* family. However, the relative abundance of *glsB*, normalised against the abundance of the single copy *recA* gene, did not show an obvious distribution pattern across *Tara* metagenome sampling sites (Fig. 5c).

In order to disentangle the multiple sources of variation likely to be driving the distribution of *glsB* in *Tara* ocean metagenomes, we employed a linear regression model [20] to test the hypothesis that genes involved in aminolipid biosynthesis, including *glsB*, are more abundant in P-depleted areas of the ocean. Data presented in Table 2 show a significant correlation between *glsB* and N* ($\left[ {NO_3^ - } \right] - 16\left[ {PO_4^{3 - }} \right]$), an indicator of the relative concentration of inorganic N and P; [20]) as well as *olsB* and N* (*p* < 0.05 and *p* < 0.001, respectively), suggesting that the relative abundance of these two genes are indeed positively correlated to the relative concentration of the inorganic nutrients N and P. In contrast, *olsF* showed no significant correlation to the relative concentration of the inorganic nutrients N and P. A close investigation of the *olsB*-N* relationship indicates that the greater slope coefficient in *olsB*, compared to *glsB*, is likely driven by the presence of SAR11 genes in the *olsB* dataset but not in the *glsB* dataset since SAR11 isolates are known to produce ornithine lipids but not glutamine lipids in response to P-depletion [5].

**Table 2:** Likelihood ratio test (LRT) comparisons of generalised linear models for aminolipid synthesis gene abundance with and without the inclusion of N* as an independent variable

					Model comparison	
Gene	Slope coefficient	Standard error	*z*-value	*p*	LRT statistic	LRT *p*
*glsB*	0.179	0.087	2.06	<0.05	4.35	<0.05
*olsB*	0.201	0.056	3.62	<0.001	13.2	<0.001
*olsF*	−0.078	0.077	−1.03	0.304	1.22	0.269

Coefficients are given for the N* term along with *z*-value and associated *p*-values for the inclusion of N* as a parameter. N* is defined as $\left[ {NO_3^ - } \right] - 16\left[ {PO_4^{3 - }} \right]$

Together, analysis of the genes involved in glutamine lipid and ornithine lipid biosynthesis in these *Tara* ocean metagenomes suggest these lipids are important in adapting to nutrient stress in abundant marine bacteria, especially the *Rhodobacteraceae*.

We next determined if the *glsB* gene is indeed actively expressed in the marine environment. Searching available metatranscriptomes in the JGI IMG database, using a stringent *e*-value cut-off of 10^−20^, we retrieved more than 100 hits, the majority of which (>95%) are classified as *Rhodobacteraceae* (Table S1). Phylogenetic analysis showed that the actively expressed *glsB* genes largely originated from pelagic *Roseobacter* strains (Figure S3), e.g. *Planktomarina temperata* RCA23 [24], *Rhodobacteraceae* sp. HIMB11 [29], and *Rhodobacteraceae* sp. SB2 [30].

## Discussion

Aminolipids are a poorly studied class of lipids, which seem to be found exclusively in bacteria [9]. Although several aminolipids have been identified in bacteria, only the biosynthesis of ornithine lipid has been characterised previously [11, 31, 32]. In this study, using the marine bacterium *R. pomeroyi* DSS-3 as a model, we characterized the *glsB* gene responsible for the first step in glutamine-containing aminolipid formation. A second gene, *olsA*, which has previously been shown to convert lyso-ornithine to ornithine lipid [32], was also required for glutamine lipid biosynthesis (Fig. 2). These findings indicate that glutamine lipid biosynthesis likely proceeds via a two-step process, analogous to the synthesis of ornithine lipid (Fig. 2). The first step in glutamine lipid biosynthesis, the *N*-acylation of glutamine with a 3-hydroxy fatty acid is mediated by GlsB. The second step, the *O*-acylation of the hydroxyl group of the first fatty acid, appears to be catalysed by the acyltransferase OlsA. OlsA can also acylate glycerol-3-phosphate to form phosphatidic acid, an intermediate in phospholipid biosynthesis [33], indicating that it has a relatively broad substrate specificity. Interestingly, lipidomics analyses of the *R. pomeroyi* mutants showed that disruption of *olsA* did not result in an accumulation of lyso-aminolipids, which might be expected to accumulate based on the proposed biosynthetic pathway (Fig. 2). However, this lack of detectable lyso-aminolipids is consistent with prior studies in *E. meliloti ΔolsA* mutant strains [34]. It would appear that these lyso-aminolipids are under tight control in the cell and rapidly degraded if they are not acylated by OlsA to form the intact aminolipid.

The specific physiological role of glutamine lipid in *Roseobacters* remains unclear. A slight growth defect was observed for the *ΔglsB* mutant of *R. pomeroyi* DSS-3, deficient in glutamine lipid but not ornithine lipid biosynthesis, in low-P medium relative to the wild type (Table 1). Interestingly, the *ΔolsA* mutant, deficient in both glutamine lipid and ornithine lipids, exhibited a more severe growth phenotype in high-P medium and ceased to grow in low-P medium (Table 1). Our data therefore strongly suggest that these aminolipids are required for normal cell function, particularly during P-deplete growth. It is likely that ornithine lipids and glutamine lipids may functionally substitute for one another, resulting in a more severe phenotype when both are removed. Repeated attempts to grow the *ΔolsA* mutant in a range of phosphate concentrations below 0.5 mM (50 μM–0.25 mM) reproduced this lack of growth (data not shown). The reason for this lack of viability in low-P medium is unclear: one explanation could be that a sufficient concentration of phosphate ions is required to stabilise the membrane in the absence of either aminolipid in the *ΔolsA* mutant. This would be analogous to the phenotype of *E. coli* mutants lacking phosphatidylethanolamine, which require divalent cations (such as Ca^2+^) for viability [35].

In contrast, the role of ornithine lipid in bacterial physiology has been studied in several model bacteria (reviewed by [10] and references therein). Previous findings of a role for this lipid in maintaining optimal amounts of *c*-type cytochromes in *Rhodobacter capsulatus* supports the view of aminolipids playing an integral role in *Rhodobacteraceae* biology [36]. However, in *E. meliloti*, a lack of ornithine lipids had a minimal impact on fitness except when P was limiting [34]. Several other bacterial strains also appear to only synthesise this lipid when grown in P-deplete medium [5, 37]. These observations suggest a model whereby aminolipids play discrete roles in different bacteria: in some strains the capacity to produce aminolipids has largely been acquired as an adaptation to P scarcity, whilst in other bacteria they play a more integral role in cell physiology, e.g., for maintaining c-type cytochrome functions [36].

Our analysis of aminolipid synthesis genes in the *Tara* metagenomes data set provided some support for this hypothesis (Table 2, Suppl. Figures S4, S5). For example, the abundance of *olsB* showed an overall positive relationship with N*, indicating that it provides a selective advantage in P-deplete conditions. This strong correlation is at least partially explained by the presence of *olsB* in SAR11 bacteria, which are known to upregulate ornithine lipid production in response to P-stress [5]. On the other hand, the two groups contributing the most to overall *olsB* abundance, the *Rhodobacteraceae* and the *Rhodospirillales* (Suppl. Table S3), showed no significant relationship with N*. Conversely, there was a significant positive correlation between *glsB* abundance and N* in the *Tara* dataset. However, the abundance of *olsF* was not influenced by N* (Suppl. Figure S4). Unlike OlsB and GlsB, which are primarily found in *Alphaproteobacteria*, OlsF is the aminolipid synthesis gene most commonly found in *Gammaproteobacteria* and *Bacteroidetes* (Fig. 5). A recent lipidomic analysis of one marine *Bacteroidetes* strain, *Dokdonia* sp. MED134, showed the presence of several aminolipid classes which comprised a substantial proportion of the lipidome even in P-replete conditions [3]. The role of aminolipids and whether *olsF* is responsible for aminolipid synthesis in these marine *Bacteroidetes* awaits to be determined.

Our genome, metagenome and metatranscriptome analyses showed that the capability to synthesise glutamine lipid appears to be highly conserved in the *Rhodobacteraceae* whereas the ability to make ornithine lipids is widespread in many ecologically important marine bacteria groups, including the abundant SAR11 clade [5] and the marine *Bacteroidetes* (Fig. 5). However, only a few studies have reported the detection of aminolipids in the marine environment, and, to the best of our knowledge, no aminolipids other than ornithine lipids have been reported in aquatic ecosystems. One such study, conducted in the Black Sea, detected ornithine lipids in deeper, anoxic water, but not at the surface [6]. The failure to detect ornithine lipids in surface waters is puzzling, given the widespread distribution of *olsB* and *olsF* in the genomes of sequenced bacteria and marine metagenomes (Fig. 5), and its presence in some strains of the widespread SAR11 clade [5, 11]. At present it is unclear whether the lack of reported aminolipids in marine surface waters reflects shortcomings in the analytical techniques used to detect lipids in these environments, or a genuine lack of these lipids. Our metatranscriptome analysis supports the notion that glutamine lipid biosynthesis occurs in marine water columns (Table S1, Figure S3), particularly in members of the numerically abundant and metabolically active pelagic *Roseobacter* clade [24, 30]. Given the widespread occurrence and expression of aminolipid biosynthesis genes in ecologically important marine bacteria, whether mass spectrometry-based lipidomics techniques have overlooked these compounds certainly warrants further investigation.

## Electronic supplementary material

Table S1

Table S2

Table S3

Supplementary figure legends

Fig S1

Fig S2

Fig S3

Fig S4

Fig S5
