# Supplementary material for: Elucidation of glutamine lipid biosynthesis in marine bacteria reveals its importance under phosphorus deplete growth in Rhodobacteraceae
Source: ISME J. 2018 Aug 14;13(1):39–49. doi: 10.1038/s41396-018-0249-z (PMC6298996; doi:10.1038/s41396-018-0249-z)
Supplement: Supplementary file 2 — Table S2 [file 41396_2018_249_MOESM2_ESM.docx]

**Table S2** Bacterial strains, plasmids and primers used in this study

| **Bacterial strains** |  |
| --- | --- |
| *Ruegeria pomeroyi* DSS-3 | Wild-type strain |
| *R. pomeroyi* Δ*glsB* | Δ*glsB* mutant |
| *R. pomeroyi* Δ*olsA* | Δ*olsA* mutant |
| *Dinoroseobacter shibae* DFL12 |  |
| *Sagittula stellate* E-37 |  |
| *Roseobacter litoralis* Och 149 |  |
| *Oceanicola batsensis* HTCC2597 |  |
| *Marinovum algicola* |  |
| *Roseobacter* sp. MED193 |  |
| *Phaeobacter gallaeciensis* DSM 26640 |  |
| *Escherichia coli* JM109 | General cloning |
| *E. coli* S17-1λ*pir* | For conjugation with *Ruegeria pomeroyi* DSS-3 |
| **Plasmids** |  |
| pGEM-T | General cloning |
| pK18*mobsacB* | Suicide vector for *Ruegeria pomeroyi* DSS-3 |
| P34S-Gm | Source of the gentamicin resistance (Gm^R^) gene cassette |
| **Primers (5’-3’)** |  |
| *ΔglsB* construction (upstream region) | Forward: CAATTCTAGAGGTGTTCCGTATTGTGGCAA  Reverse: CAATGGATCCGCTCAACAGTATGGCATCCG |
| *ΔglsB* construction (downstream region) | Forward: CAATGGATCCGTTTACCGGGCTTGAGATCG  Reverse: CAATTCTAGAGCCTCGATATCCAGATGGTTG |
| Confirmation of *ΔglsB* mutant | Forward: AGGTAACGCTGTTTTGTGGCAG  Reverse: TCATCCTCCCAGATCTTGTCGC |
| *ΔolsA* construction (upstream region) | Forward: tatgacatgattacgaattcgagctcggtaGAATATGACCTTGGCGTGC  Reverse: cgaacaggcttatgtACTCCGCGTGCCAGTACC |
| *ΔolsA* construction (downstream region) | Forward: ttgtcacaacgccgcGGACGGGTCGAACTGATC  Reverse: cacgacgttgtaaaacgacggccagtgccaCGAACGGATCGTACTGCTT |
| Δ*olsA* construction (Gm^R^ cassette) | Forward: actggcacgcggagtACATAAGCCTGTTCGGTTCG  Reverse: cagttcgacccgtccGCGGCGTTGTGACAATTT |
| Confirmation of *ΔolsA* mutant | Forward: GTGGGCGTCTATCGTCTGTT  Reverse: AACGGTTCCTCGAGACCTTC |
