# Supplementary material for: Elucidation of glutamine lipid biosynthesis in marine bacteria reveals its importance under phosphorus deplete growth in Rhodobacteraceae
Source: ISME J. 2018 Aug 14;13(1):39–49. doi: 10.1038/s41396-018-0249-z (PMC6298996; doi:10.1038/s41396-018-0249-z)
Supplement: Supplementary file 3 — Table S3 [file 41396_2018_249_MOESM3_ESM.docx]

**Supplementary Table S3** Microbial groups used as co-variants in models of aminolipid synthesis gene abundance

| **Microbial groups** | ***olsB*** | ***glsB*** | ***olsF*** |
| --- | --- | --- | --- |
| *Rhodobacteraceae* | Yes | Yes |  |
| *Rhodospirillales* | Yes |  |  |
| SAR11 | Yes |  |  |
| *Rhizobiales* | Yes |  |  |
| Other *Alphaproteobacteria* | Yes |  |  |
| *Gammaproteobacteria* | Yes |  | Yes |
| *Bacteroidetes* |  | Yes | Yes |
| Others | Yes |  |  |
