# Supplementary material for: Elucidation of glutamine lipid biosynthesis in marine bacteria reveals its importance under phosphorus deplete growth in Rhodobacteraceae
Source: ISME J. 2018 Aug 14;13(1):39–49. doi: 10.1038/s41396-018-0249-z (PMC6298996; doi:10.1038/s41396-018-0249-z)
Supplement: Supplementary file 4 — Supplementary figure legends [file 41396_2018_249_MOESM4_ESM.docx]

**Figure S1 a)** Growth of wild type and mutant *Ruegeria pomeroyi* DSS-3 in medium with high and low phosphate. Wild type (WT) *R. pomeroyi* DSS-3 and strains deficient in *glsB* and *olsA* were grown in a defined medium with either 5 mM (circles) or 0.5 mM (triangles) of added phosphate. Points represent the mean of three biological replicates while the shaded ribbons cover the region within one standard deviation of the mean. **b)** Quantification of the phospholipid phosphatidylethanolamine (PE) in the wild type and the Δ*glsB* mutant under high (5 mM) and low (0.5 mM) phosphate conditions. The internal standards C17:0/C17:0 diheptadecanoyl-PE were purchased from Avanti Ltd and added to lipid extracts of WT *Ruegeria pomeroyi* DSS-3 and mutant cells before analysing using HPLC-MS. Quantification of PE was expressed as the ratio of PE/diheptadecanoyl-PE. The dominant PE species in *R. pomeroyi* DSS-3 is C36:2 PE (m/z 742). Measurements were carried out in three biological replicates each with three technical replicates and the error bars represent standard deviation.

**Figure S2** Phylogeny of GlsB and OlsB homologues from the genomes of the strains used to construct the phylogeny in Figure 3. Protein sequences were aligned using Metaligner (Section 2.5.2) and a maximum likelihood phylogenetic tree inferred using RaxML with 100 bootstrap replicates. Nodes with >49% bootstrap support are indicated with circles (grey: 50 – 69%; black: >70%). Edges are coloured according to the synteny of the genes on their descendant leaves, as indicated by the inset schematics. Genes in red are downstream of a nearby predicted BamE while blue indicates that the genes are immediately upstream of a predicted OlsA. Leaves in black matched neither of these criteria. Two sequences, Ga0056889_10411 from *Falsirhodobacter* sp. alg1 and Rhd02DRAFT_00243 from *Marinosulfonomonas* sp. PRT002, both placed within the blue OlsB cluster, could not be classified based on synteny since they were located at the end of a contig with no genes downstream of them.

**Figure S3** Phylogenetic analyses of GlsB sequences retrieved from marine metatranscriptomics datasets available from the Joint Genome Institute Integrated Microbial Genomes & Microbiomes database (as of 12 June 2018). A total of 428 datasets were searched using the characterized GlsB (SPO2489) from *Ruegeria pomeroyi* DSS-3 as the query (BlastP *e*-value 10^-20^). This returned 131 GlsB homologs (Table S1). Multiple sequence alignment was performed with sequences >140 amino acid in length using the MEGA 7 package and a phylogenetic tree was constructed using the neighbour-joining method with 500 bootstraps.

**Figure S4** Scatterplots and generalised linear model (GLM) fits showing the relationship between aminolipid synthesis gene counts and N* in the *Tara* metagenomes. **a, c** and **d** show GLM fits for the relationship between N* and OlsB, GlsB and OlsF, respectively. All other model parameters were held constant at their mean values. N* significantly predicted the abundances of OlsB and GlsB but not OlsF. Scatterplots show the original data points. **b**) Lines showing the fitted relationships between N* and OlsB counts for the microbial groups for which the regression slope was significantly greater than zero. Points show the original data values. Also shown are the original values for SAR11 OlsB. The horizontal bar below the data shows the range of N* values at which SAR11 OlsB was found. Counts for aminolipid synthesis genes were normalized against counts for RecA.

**Figure S5** Global maps of the abundance of OlsB (**a**) and OlsF (**b**), normalized to the abundance of RecA in the Tara metagenome dataset. Only surface water samples (collected at 5 m depth) are shown. Grey circles indicate no sequences corresponding to that gene were detected in the sample.
