# Supplementary figures and images for: Elucidation of glutamine lipid biosynthesis in marine bacteria reveals its importance under phosphorus deplete growth in Rhodobacteraceae
Source: ISME J. 2018 Aug 14;13(1):39–49. doi: 10.1038/s41396-018-0249-z (PMC6298996; doi:10.1038/s41396-018-0249-z)

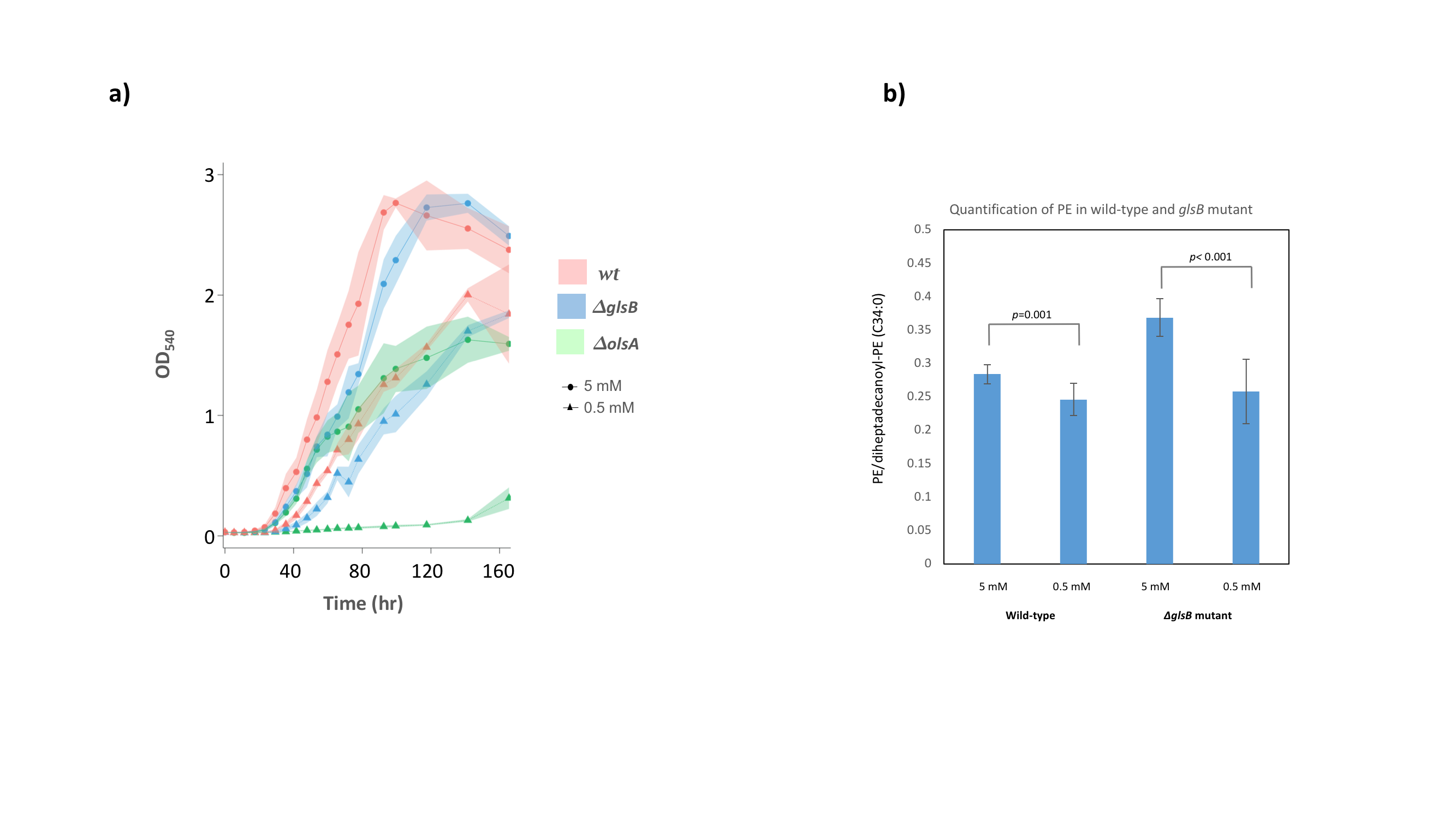

Supplement: Supplementary file 5 — Fig S1 [file 41396_2018_249_MOESM5_ESM.tif]

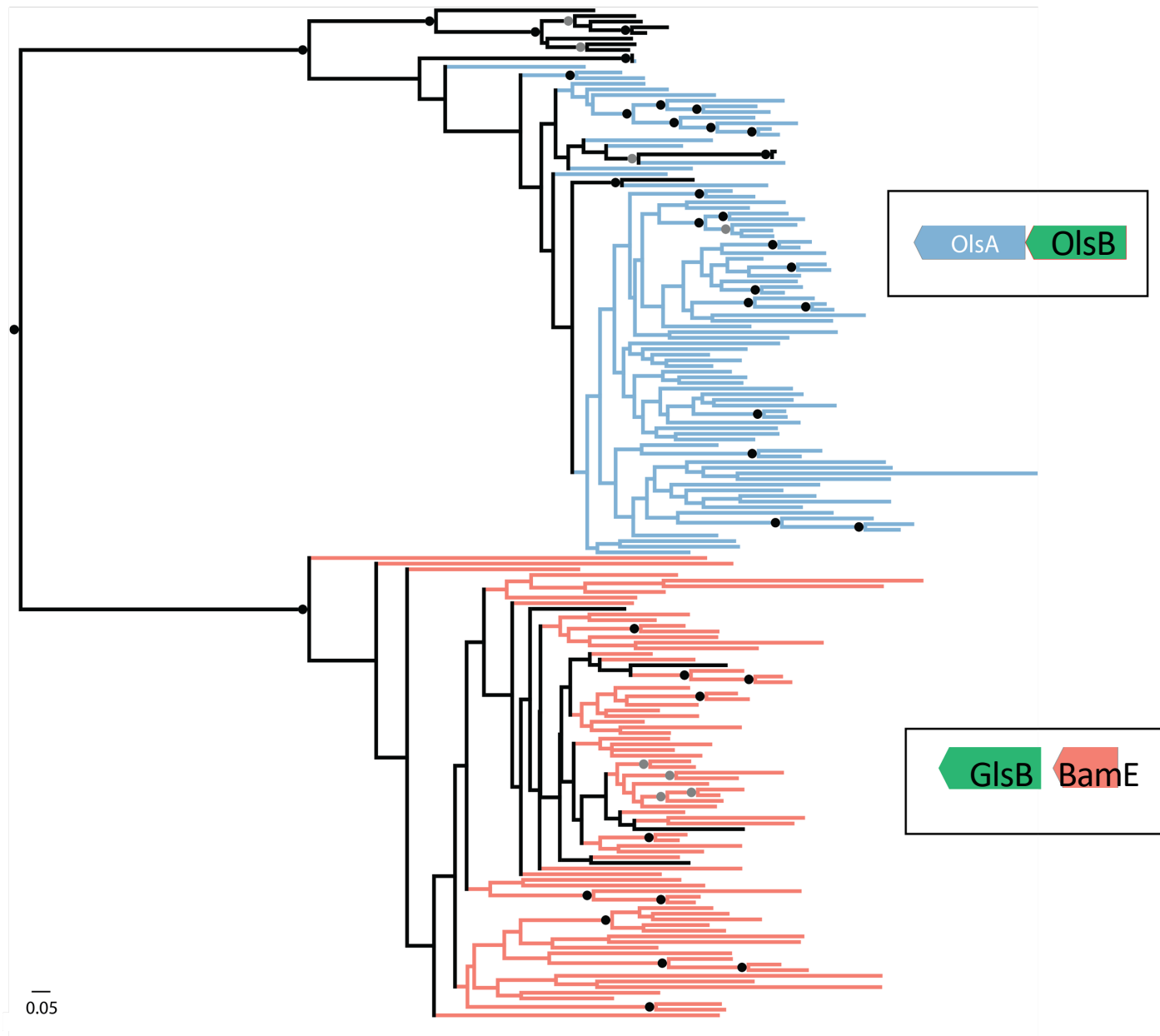

Supplement: Supplementary file 6 — Fig S2 [file 41396_2018_249_MOESM6_ESM.pdf]

Rhodobacteraceae bacterium SB2

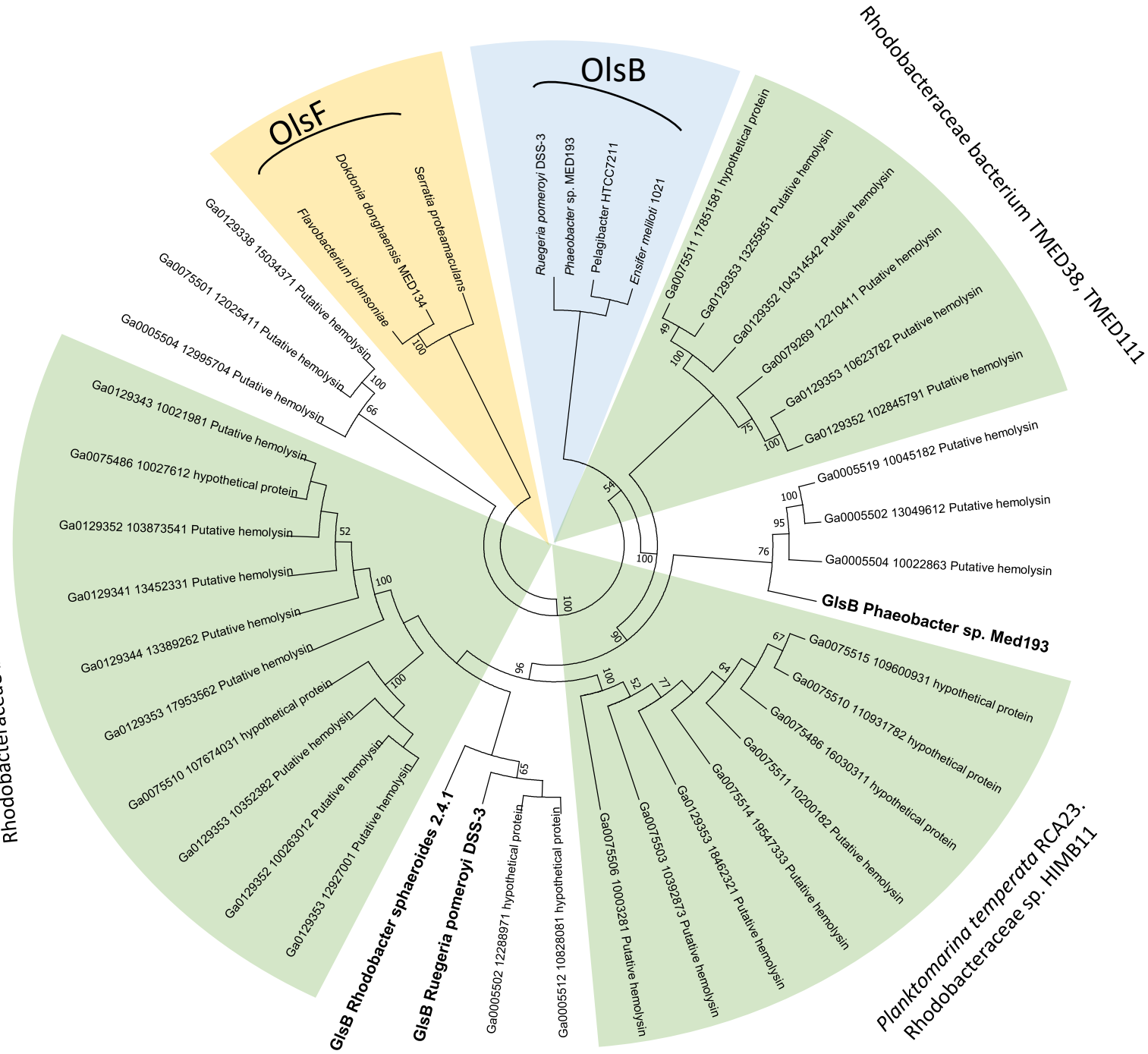

Supplement: Supplementary file 7 — Fig S3 [file 41396_2018_249_MOESM7_ESM.pdf]

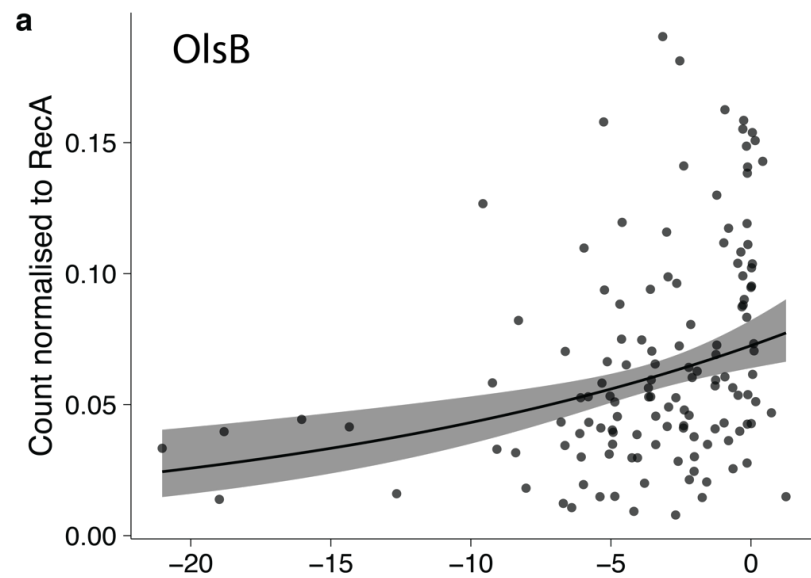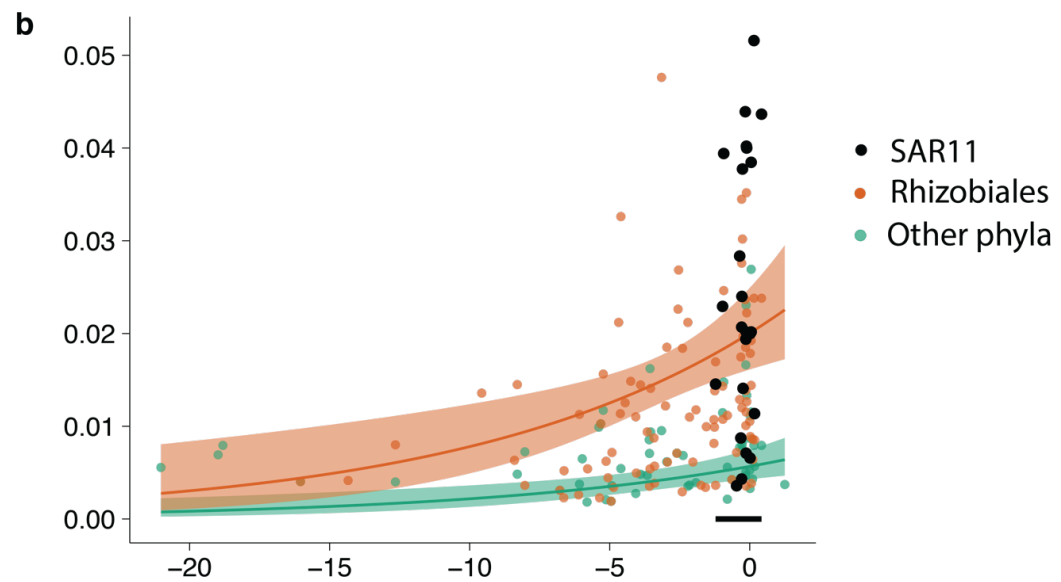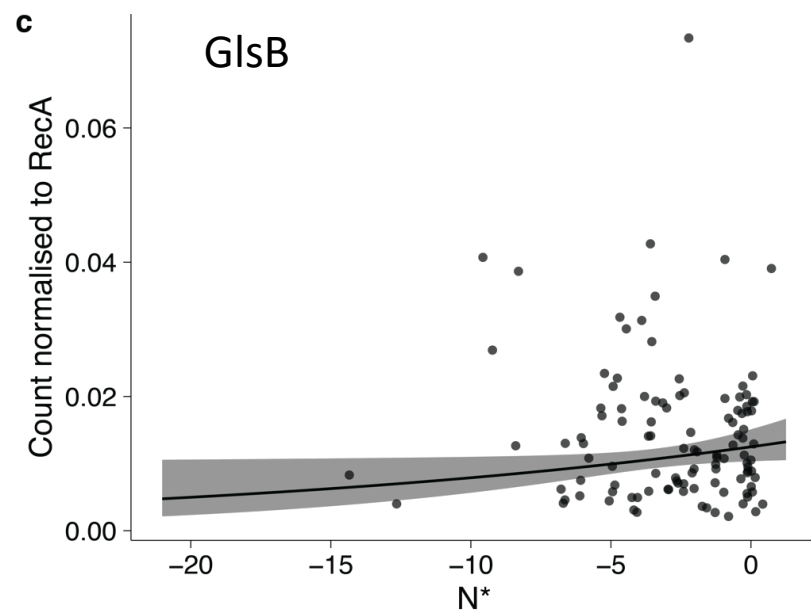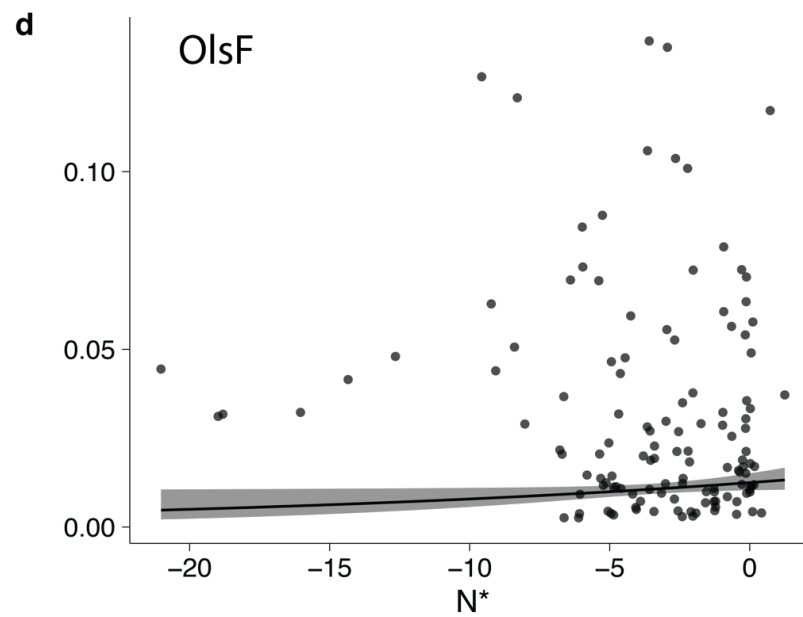

Supplement: Supplementary file 8 — Fig S4 [file 41396_2018_249_MOESM8_ESM.pdf]

a)

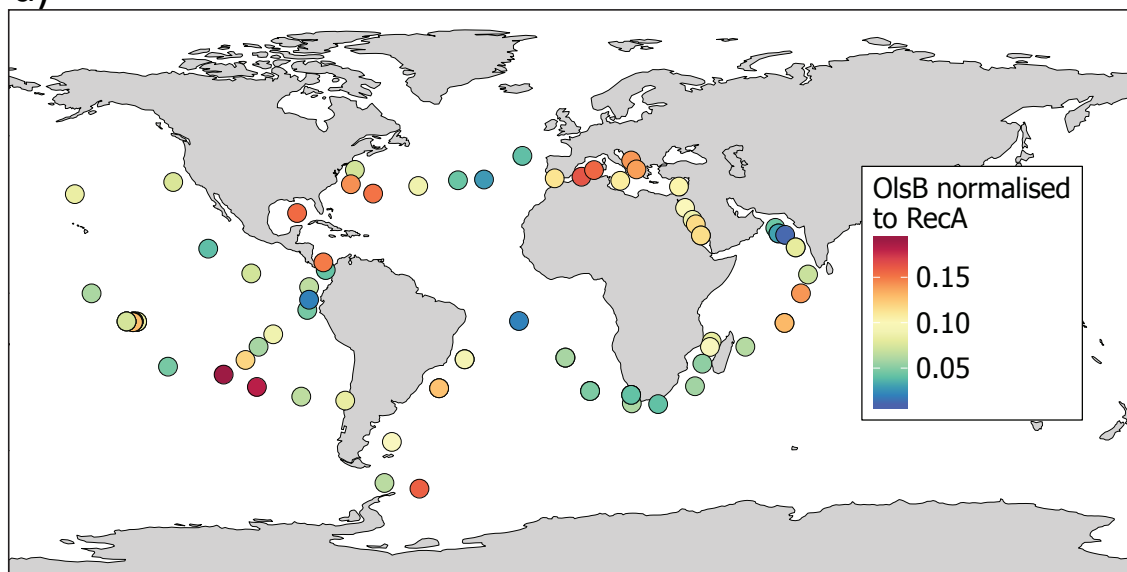

b)

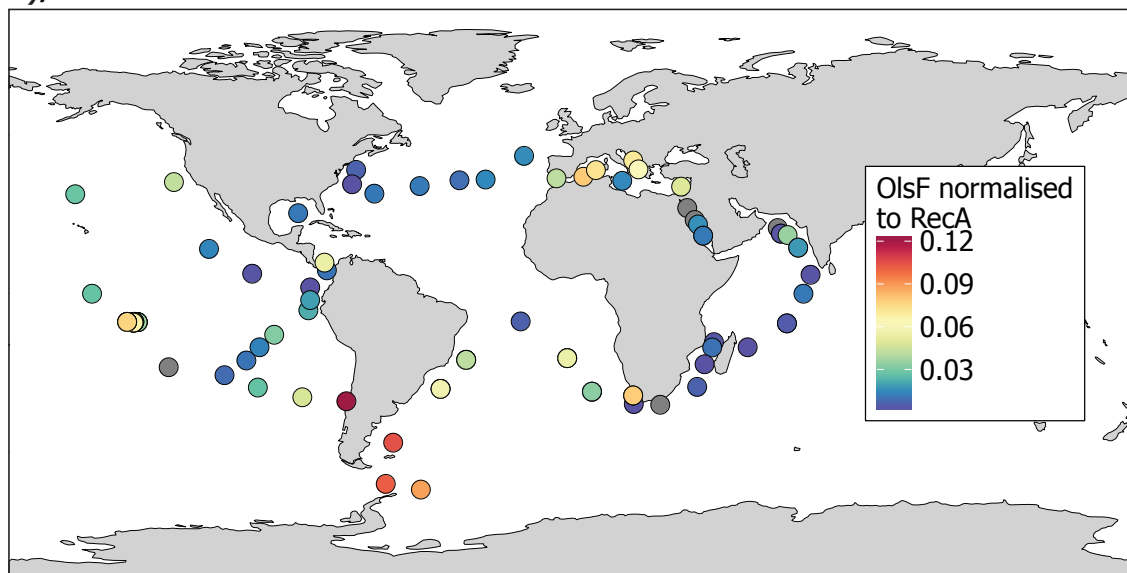

Supplement: Supplementary file 9 — Fig S5 [file 41396_2018_249_MOESM9_ESM.pdf]
